# Posttonsillectomy Bacteremia and Comparison of Tonsillar Surface and Deep Culture

**DOI:** 10.1155/2014/161878

**Published:** 2014-10-22

**Authors:** Mahmood Shishegar, Mohammad Javad Ashraf

**Affiliations:** ^1^Department of Otolaryngology, Head and Neck Surgery, Shiraz University of Medical Sciences, Zand Boulevard, Shiraz 7134845794, Iran; ^2^Department of Pathology, Shiraz University of Medical Sciences, Zand Boulevard, Shiraz 7134845794, Iran

## Abstract

*Objective.* This study aimed to identify the microorganisms of surface and depth of tonsils and whether these microorganisms bring the menace of bacteremia during tonsillectomy in the children under surgery. *Materials and Methods.* The culture specimens were taken from surface and depth of tonsil from the patients suffering from chronic tonsillitis at the time of operation. Also, 10 mL venous blood samples were taken 5 minutes before and after the operation for microbiological study. *Results.* According to the results, 112 (76.1%) and 117 (79.6%) cultures from surface and depth of tonsils represented multiple microorganisms, respectively. Besides, staphylococci coagulase positive was the most common organism in both surface and depth of tonsils. None of the preoperation blood cultures were positive, while 3 postoperation blood cultures (2.1%) were positive. Staphylococci coagulase negative and alpha hemolytic streptococcus were detected in 2 cases (1.4%) and 1 case (0.7%), respectively. *Conclusion.* In the present study, the two cultured sites were almost similar regarding the types of isolated microorganisms. Our results suggested that bacteremia might occur after tonsillectomy. Therefore, to avoid the possible dramatic outcomes after tonsillectomy, pre- and postoperation attendances are essential.

## 1. Introduction

Tonsils are important structures of the immune system and tonsillectomy is a very common surgery in childhood [1]. Leukocytes infiltrations, swelling, necrosis, and surface ulceration in tonsils may create in acute bacterial infection of the tonsils [2]. Thus, antibiotic therapy may be sufficient in acute infections. Nevertheless, inappropriate treatment against the microorganisms or entering low levels of antibiotics into depth of tonsil leads to continuation of the infection and recurrence of tonsillitis [3]. Therefore, tonsillectomy is the choice treatment in chronic and recurrent cases [4]. Bacteremia and local infection can occur after tonsillectomy [5]. Bacteremia in the patients who have experienced tonsillectomy due to chronic tonsillitis may lead to intense outcomes, especially in those who are faced with the risk of cardiovascular infection [6, 7]. Some studies have shown that the bacterial pathogens related to tonsillitis inhabited the surface and depth of the tonsil tissue [8]. Moreover, a large number of studies have presented similarities between the types of isolated microorganisms from the surface and depth of tonsil, but the degree of likeness has been very different in these studies [6, 9–11]. The cultures taken from surface of tonsil cannot display the real existing pathogens; thus, cultures from the depth of tonsil are necessary for identification of tonsil microbiology and selection of the appropriate treatment strategy [6, 9–12]. The present study aims to evaluate the microorganisms of surface and depth of tonsil in the patients with chronic tonsillitis. It also aims to determine whether these microorganisms bring the menace of bacteremia during tonsillectomy.

## 2. Materials and Methods

The present study was conducted on 147 patients selected from the childhood group whose age ranged from 3 to 15 years. The patients were admitted to Khalili General Hospital from September 2011 to September 2012. The patients' indications for tonsillectomy were suffering from recurrent chronic tonsillitis and not having received antibiotics for the last 48 hours.

All the procedures were performed under general anesthesia with endotracheal intubation which was maintained by inhalation anesthesia. Tonsillectomy was done by dissection routine method.

In this study, 10 mL venous blood samples were taken 5 min before and after the operation and were transmitted to Tryptic Soy Broth (TSB) culture media.

The culture samples from the surface of tonsils were taken by sterile swab. After tonsillectomy, the tonsils were rinsed out using sterilized physiologic normal saline and cauterized by heated scalpel. Afterwards, the tonsils from the cauterized areas were divided into two parts and the samples were taken from the core of the tonsils using a sterile swab. These swabs were put at thioglycollate broth bottles. Then, all the samples were transported to the laboratory as soon as possible and the specimens were incubated at 37°C for 24 h. After 24 hours of incubation, subcultures on blood agar from growing microorganism were prepared. The isolated bacteria were gram stained and microscopically investigated. Then, they were identified biochemically by API 20E strip test (bioMerieux-vitek, Hazelwood, MO) [13].

## 3. Results

In the present study, 80 subjects (54.5%) were male and 67 (45.5%) were female. The patients' age ranged from 3–15 years (mean age: 5.3 years). Age and sex distribution of the patients has been presented in Table 1.

The growth of microorganisms from the surface and depth of the tonsils was positive in 112 (76.1%) and 117 (79.6%) patients, respectively.

In addition, a total of 395 bacteria isolated from both surface and deep cultures, as mixed types of bacteria, were isolated in the same case. Our study results showed that staphylococci coagulase positive (26%) followed by *β* nonhemolytic streptococci (16.5%) and normal flora (15%) was the most common aerobic organism. On the other hand, staphylococci coagulase negative (14%), hemolytic streptococci (12.5%), and *α* hemolytic streptococci (10%) had less frequently grown from the surface of the tonsils.

Considering the tonsillar deep cultures, staphylococci coagulase positive (31.5%) was the most common isolate followed by normal flora (18.5%), staphylococci coagulase negative (15%), *α* hemolytic streptococci (13%), and *β* nonhemolytic streptococci (10%) (Table 2).

The results of blood cultures showed no growth on the blood cultures taken before tonsillectomy, while 3 patients (2.1%) had positive cultures after the operation. Among these cases, staphylococci coagulase negative and alpha hemolytic streptococcus were detected in 2 cases (1.4%) and 1 case (0.7%), respectively.

## 4. Discussion

Tonsillectomy is a common surgical approach generally applied for Sleep-Disordered Breathing (SDB) and recurrent infections of tonsils, especially in children [14]. The results of the studies performed over the last decade have shown SDB as the most common symptom of pediatric tonsillectomy, predominantly in younger children [15, 16].

Based on the results of various studies, pathogenic bacteria were detected in both surface and depth of the tonsil tissue cultures of a considerable number of patients involved with tonsillitis [11].

In present study, staphylococci coagulase positive was the most prevalent bacterium isolated from the tonsillar culture. Similarly, in the study by Ozturkcan et al., streptococci and staphylococci were the predominant isolates, while anaerobic organisms were the least common ones [17]. Besides, Surow et al. showed that* Staphylococcus aureus* was rarely reflected on the surface culture [18]. These findings were consistent with the results of our study.

However, our present findings were not similar to the result of some comparative studies. For instance, Loganathan et al. suggested that* Staphylococcus aureus* and Group A *β* hemolytic streptococci were the most common isolated bacteria from the tonsillar culture [19]. In addition, Mitchelmore et al. showed that anaerobes were the major isolated bacteria of the tonsil surface and core [20]. Kielmovitch et al. and Surow et al. also revealed a high incidence* of Haemophilus infleunzae* in the tonsillar cultures [18, 21].

Brodsky et al. described that the bacteria isolated from tonsillar deep tissue were more similar to the isolated bacteria from the tonsillar surface [22]. Furthermore, other studies implied similarities among the types of isolates from surface and depth of tonsils tissues; nevertheless, the degree of likeness varied from 32% [9] to 38% [11] and 50% [10] in different studies. In the present study, the majority of the isolated organisms from the tonsillar surface were correlated with those isolated from the tonsillar deep specimens. Nevertheless, significant differences were found between the surface and deep tissue cultures regarding *β* nonhemolytic streptococci and hemolytic streptococci.

Bacteremia may develop as a result of tonsillectomy. Bacteria can diffuse through the veins in the tonsillar tissue or through the open wound margins [23]. The traction before tonsillectomy may also have a role in bacterial diffusion [24]. Posttonsillectomy bacteremia is a well-recognized etiological factor in streptococcal endocarditis [24]. Kocatürk et al. suggested that transient bacteremia may originate from the tonsillar core tissue [25]. In the present study, however, coagulase negative staphylococcus and alpha hemolytic streptococcus that were two isolated bacteria from the blood cultures had almost equal ratios of isolation from the surface and depth of the tonsils.

In the current study, the less prevalent organisms isolated from the blood cultures were comparable with the results of the study by Olina et al. showing streptococci (21.5%) and staphylococci (9.8%) as the common isolated organisms [26]. In line with our findings, Kaygusuz et al. also recognized alpha-hemolytic streptococcus in one case [6]. On the other hand, some studies displayed different organisms. For instance, Francois et al. showed that* Haemophilus influenzae* were the prevalent isolated bacteria after tonsillectomy, while similar to our results, alpha-hemolytic streptococci were the second common isolated bacteria [12]. In the research by Gaffney et al.* Haemophilus influenzae* and* Streptococcus viridans* were isolated from 36.4% and 9% of the blood cultures, respectively [27]. On the other hand, Soldado et al. reported that* Haemophilus influenzae* and* Streptococcus viridans* were isolated from 56% and 36.5% of the positive cultures, respectively [24]. In another study,* Pseudomonas aeruginosa* was detected in 28.6% of the positive cultures [28].

In some studies, bacteremia was produced in 13%, 22%, 25%, and 40.1% of all the patients after tonsillectomy [6, 24, 25, 29]. In our study, on the other hand, this rate was found as 2.1%.

The differences among the rates of bacteremia before and after tonsillectomy may be attributed to various factors, such as different blood culture gathering times, blood culture methods, amount of bleeding during the surgery, and history of recurrent Acute Otitis Media (rAOM) or recurrent Tonsillopharyngitis (rTF) in the patient [30–32].

In general, blood cultures can be collected during tonsillectomy [12], 2 minutes after the removal of the second tonsil [32], and in the postoperative period [6]. In our study, blood culture was taken within 5 minutes before starting and after completion of tonsillectomy.

Yildirim et al. showed that timing of culture sampling was important for bacteremia detection. They investigated two groups of blood cultures taken within two minutes and 15 minutes after tonsillectomy and the difference between the two groups was statistically significant (*P* < 0.05) [5].

Posttonsillectomy bacteremia values may also be influenced by the surgical techniques. Gaffney et al. reported that the guillotine method compared to the dissection method showed a lower posttonsillectomy bacteremia rate [27]. Inconsistently, in another study, the rate of bacteremia with the guillotine technique and the dissection technique was 60% and 19%, respectively [26]. Conversely, Walsh et al. showed no statistically significant differences between the two procedures regarding the occurrence of bacteremia [32].

Furthermore, Koc et al. concluded that bacteremia was more frequent after tonsillectomy with greater amount of bleeding during the surgery [31]. Also, Esposito et al. showed that bacteremia was significantly more associated with adenotonsillectomy compared to adenoidectomy and was significantly more frequent in the patients with a history of rAOM or rTF [30].

## 5. Conclusion

Various studies have revealed different results regarding the types and similarity of the isolated microorganisms from surface and deep tonsil tissues and the rate of bacteremia. In the present study, the growth of the same pathogen bacteria in blood and tonsillar tissue cultures recommended that bacteremia could originate from the tonsillar tissue bacteria. Thus, antibiotic therapy is necessary, especially in the patients suffering from chronic tonsillitis with cardiovascular diseases. Of course, conducting larger case-series which reflect the microbiological profiles of the Iranian people is necessary to define the choice of antibiotics for prophylaxis management.

## Figures and Tables

**Table 1:** Age and sex distribution of the patients.

	3–5 years		5–10 years		10–15 years		Total	
	Number	Percentage	Number	Percentage	Number	Percentage	Number	Percentage
Male	41	27.8	38	25.8	1	0.7	80	54.5
Female	35	23.8	32	21.7	0	0	67	45.5

Total	76	51.7	70	47.6	1	0.7	147	100

**Table 2:** The various organisms isolated from the surface and depth of the tonsils of the patients with chronic tonsillitis.

Microorganisms	Isolates from the surface	Isolates from the deep	Surface and deep
	*N* (%)	*N* (%)	*N* (%)
SC +	61 (26)	51 (31.5)	112 (28.5)
NF	35 (15)	30 (18.5)	65 (16.5)
SC −	33 (14)	24 (15)	57 (14.5)
BNHS	39 (16.5)	16 (10)	55 (14)
AHS	24 (10.5)	21 (13)	45 (11.5)
HS	29 (12.5)	12 (7.5)	41 (10.5)
Enterobacter	3 (1.4)	3 (1.8)	6 (1.5)
Pseudomonas	3 (1.4)	2 (1.2)	5 (1)
GABHS	3 (1.4)	2 (1.2)	5 (1)
Yeast	2 (0.8)	1 (0.6)	3 (0.8)
*S. aureus *	1 (0.4)	0 (0)	1 (0.2)

Total	233 (100)	162 (100)	395 (100)

SC +: staphylococci coagulase positive, NF: normal flora, SC −: staphylococci coagulase negative, BNHS: *β* nonhemolytic streptococci, AHS: *α* hemolytic streptococci, HS: hemolytic streptococci, and GABHS: Group A *β* hemolytic streptococci.
